# Body mass index is associated with miscarriage rate and perinatal outcomes in cycles with frozen-thawed single blastocyst transfer: a retrospective cohort study

**DOI:** 10.1186/s12884-022-04443-2

**Published:** 2022-02-11

**Authors:** Yu Zheng, Xiyuan Dong, Biao Chen, Jun Dai, Wei Yang, Jihui Ai, Lei Jin

**Affiliations:** 1 Reproductive Medicine Center, Tongji Hospital of Tongji Medical College of Huazhong University of Science and Technology, Wuhan, 430030 China; 2 Department of Gynecology and Obstetrics, Tongji Hospital of Tongji Medical College of Huazhong University of Science and Technology, Wuhan, 430030 China

**Keywords:** Body mass index, Live birth, Maternal outcomes, Neonatal outcomes, Frozen-thawed embryo transfer

## Abstract

**Background:**

The association between body mass index (BMI) and IVF cycle outcomes remain inconclusive. In addition, the impact of BMI on perinatal outcomes has been less well-studied. The aim of this study was to assess the effects of BMI on pregnancy outcomes, as well as maternal and neonatal outcomes.

**Methods:**

This was a retrospective cohort study on 10,252 frozen-thawed cycles with single blastocyst transfer between January 2016 and December 2019. Patients were divided into four groups: underweight (< 18.5 kg/m^2^), normal-weight (18.5–24 kg/m^2^), overweight (24–28 kg/m^2^), and obesity (≥ 28 kg/m^2^), according to the Chinese classification. Multivariate logistic regression and multivariate general linear model were used for statistical analysis.

**Results:**

The rates of live birth and clinical pregnancy were comparable among groups. Miscarriage rate was higher in the obese women than that in the normal controls (27.51 vs. 20.91%, aOR = 1.453 (1.066–1.982)). Using the normal-weight women as reference, the underweight women had lower incidences of preterm birth (6.97 vs. 11.19%, aOR = 0.611 (0.422–0.884)), macrosomia (4.90 vs. 8.65%, aOR = 0.544 (0.353–0.837)) and large-for-gestational age (LGA, 11.18 vs. 16.54%, aOR = 0.643 (0.477–0.866)); the overweight women had higher prevalence of gestational diabetes (6.56 vs. 3.82%, aOR = 1.744 (1.232–2.468)), hypertension (4.42 vs. 2.32%, aOR = 1.822 (1.186–2.800)), macrosomia (12.93 vs. 8.65%, aOR = 1.596 (1.240–2.054)) and LGA (23.22 vs. 16.54%, aOR = 1.549 (1.270–1.890)); the obese women had higher incidences of preterm birth (16.87 vs. 11.19%, aOR = 1.646 (1.068–2.536)), cesarean delivery (93.98 vs. 87.91%, aOR = 2.078 (1.083–3.987)), gestational hypertension (4.82 vs. 2.32%, aOR = 2.138 (1.005–4.547)), macrosomia (14.88 vs. 8.65%, aOR = 1.880 (1.192–2.964)) and LGA (25.60 vs. 16.54%, aOR = 1.764 (1.218–2.555)).

**Conclusions:**

BMI has no significant effect on the chance of pregnancy or live birth, but obesity increases the risk of miscarriage. Underweight is associated with better maternal and neonatal outcomes, while overweight and obesity are associated with worse maternal and neonatal outcomes.

**Supplementary Information:**

The online version contains supplementary material available at 10.1186/s12884-022-04443-2.

## Background

The overweight and obesity have become a global problem of public health. In the United States, around 34% women of reproductive age are overweight and 26% are obese [1]. In China, the prevalence of overweight and obese are 34.3% and 16.4%, respectively [2]. The body mass index (BMI) is commonly used for diagnosis of overweight, obesity, as well as underweight. A greater BMI is associated with reduced fecundity and infertility [3, 4]. The relevant mechanisms may attribute to impaired ovulation activity, compromised endometrial receptivity or both. Furthermore, it has been reported that increased BMI has adverse effects on pregnancy outcomes in the setting of IVF. Overweight and obese women require higher doses of gonadotropins and longer duration of stimulation, but obtain fewer oocytes [5] and lower rates of pregnancy and live birth [6]. In addition, there is mounting evidence that increased BMI has deleterious effects on obstetric complications (such as miscarriage, preterm birth, gestational hypertension, gestational diabetes [7]), neonatal outcomes (such as birth weight [8], congenital anomalies [9], neonatal death [10]), and offspring health [11].

Compared with studies on overweight and obesity, IVF outcomes of underweight women is less well-studied. Some underweight women are recommended to gain weight before initiation of IVF; however, it lacks clinical evidence to support this practice. The relationship of underweight and pregnancy outcomes would be especially relevant to Chinese women, because they have higher rates of underweight than western counterparts. There is also a concern on worse obstetric outcomes of underweight women who may have preconception undernutrition status.

Previous studies have evaluated the impact of BMI on IVF cycle outcomes; however, several factors complicate the nature of it. First, the results were conflicting on this issue. Early studies showed lower CPRs and LBRs, but higher miscarriage rates in women with increased BMIs [5, 12]. However, more recent studies found no significant difference in the rate of pregnancy, live birth or miscarriage among patients with different BMI categories [13, 14]. Second, most prior studies included only fresh cycles. Frozen-thawed embryo transfer (FET) has been increasingly used, but there are limited data available on the association between BMI and FET cycle outcomes. Finally, most studies included Caucasia women, and BMI ranges of 25–30 and ≥ 30 kg/m^2^ were used for the diagnosis of overweight and obesity, according to the WHO recommendation. In general, Chinese women are relatively short and lean, and very few of them have a BMI > 30 kg/m^2^. Therefore, the definitions of overweight and obesity in China were 24–28 and ≥ 28 kg/m^2^ [2, 15]. There is a severe lack of clinical data on the association between BMI and IVF outcomes in Chinese population.

Studies using oocytes donation suggest that the adverse effect of increased BMI occurs at the level of oocyte and subsequent embryo [16]. Indeed, animal models have shown that obesity impairs the folliculogenesis, and thus results in a decreased oocyte/embryo development potential [17]. Studies on human oocytes have shown that obesity results in alteration of ovarian follicular proteome and metabolome [18], and then leads to spindle anomalies and nonaligned chromosomes in oocytes [19]. Deleterious effects of increased BMI on oocytes may persist during the early embryo development, impair the formation of blastocyst, and decrease the chance of pregnancy. Blastocyst culture is a useful tool of screening for embryos with better competence. Therefore, we hypothesized that blastocyst vs. cleavage-stage embryo transfer may elevate the IVF success rates in women with greater BMIs.

The objective of this study was first, to assess the effects of BMI on pregnancy outcomes, as well as maternal and neonatal outcomes; and second, to verify our hypothesis that whether blastocyst transfer improves cycle outcomes in women with greater BMIs.

## Methods

### Study population

The frozen-thawed cycles with single day 5 blastocyst transfer at our center between January 2016 and December 2019 were included. The exclusion criteria were preimplantation genetic testing (PGT), uterine malformations, gametes donation /freezing, significantly varied BMI measured at transfer from that measured in the fresh cycle, and missing data of important variables. All patients were followed up to the end of pregnancy. In total, the data of 10,252 cycles were extracted for the final analysis (Additional Fig. 1). This study was approved by the Institutional Review Board (IRB) of Tongji Hospital. The data were collected from the local database. All patients in this study gave written consent regarding the inclusion of data pertaining to them. The information was anonymously processed before any analysis.

**Fig. 1:**
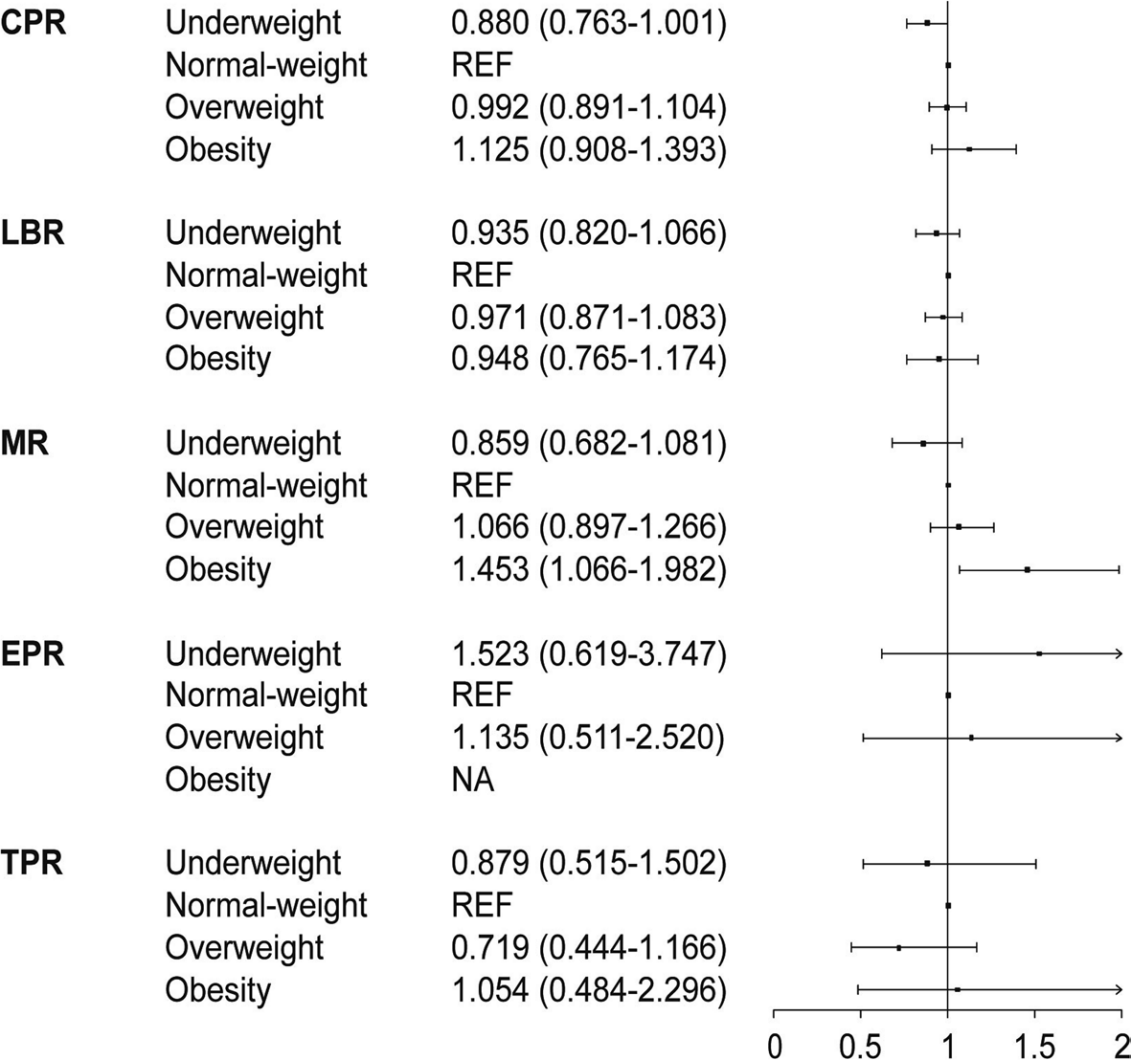
Forest plot of pregnancy outcomes according to BMI categories. *CPR* clinical pregnancy rate, *LBR* live birth rate, *MR* miscarriage rate, *EPR* ectopic pregnancy rate, *TPR* twin-pregnancy rate, *REF* reference, *NA* not applicable

To clarify the effect of embryo stage (blastocyst vs. cleavage-stage embryo) on the pregnancy outcomes of women with different BMI categories, we analyzed another cohort of 2453 cycles with single cleavage-stage embryo transfer.

The patients were divided into four groups according to the criteria of Prevention and Control of Overweight and Obesity for Chinese Adults: a guideline from Working Group on Obesity in China [15]: (1) underweight, BMI < 18.5 kg/m^2^; (2) normal-weight, BMI 18.5–23.9 kg/m^2^; (3) overweight, BMI 24.0–27.9 kg/m^2^; and (4) obesity, BMI ≥ 28 kg/m^2^.

### Endometrial preparation

The endometrial preparation was performed using a natural cycle (NC) protocol, an artificial cycle (AC) protocol, or a downregulation combined with AC (DR + AC) protocol. Regarding the NC protocol, serial trans-vaginal ultrasound scans were performed until the endometrial thickness reached ≥ 8 mm or approximated the level in the stimulated cycle. The timing of ovulation was estimated by a combined analysis of ultrasound results, the LH level and the P level. Regarding the AC protocol, E2 valerate tablets (PROGYNOVA, Bayer, Germany) were administered at 2 mg/d on day 2–4, 4 mg/d on day 5–7, and 6 mg/d on day 8–11. Serial ultrasound scans were performed from day 11–12. The dosage was adjusted based on the endometrial thickness. When the endometrial thickness reached ≥ 8 mm or approximated the level in the stimulated cycle, 40 mg intramuscular P (P injection, Xianju, China) and 20 mg oral dydrogesterone (Duphaston, Abbott, Netherlands) were used to transform the endometrium. Regarding the DR + AC protocol: a depot GnRH-a, leuprorelin acetate (BEIYI, Lizhu, China) 3.75 mg was subcutaneously administered on the second day of menstruation. Oral estrogen was administrated on the 28th day after leuprorelin injection. The following course was similar to the AC protocol.

### Embryo vitrification, warming and transfer

Embryo vitrification and warming were performed as previously described [20]. The embryos were vitrified within 2 h after scoring. The entire vitrification procedure was performed at room temperature (22–25 °C). The embryos were equilibrated in equilibration solution (ES; Vitrification kit, Kitazato, Japan), containing 7.5% ethylene glycol and 7.5% dimethylsulfoxide (DMSO), for 5–10 min. The embryos were then transferred into vitrification solution (VS; Vitrification kit, Kitazato, Japan), which contained 15% ethylene glycol, 15% DMSO, and 0.5 mol/L sucrose, and they were subsequently loaded onto the surface of a Cryotop System (Kitazato, Japan) within 40–60 s. They were then immediately submerged in liquid nitrogen.

On the day of transfer, embryos were warmed at room temperature (22–25 °C). They were transferred to thawing solution (TS; Vitrification kit, Kitazato, Japan), which contained 1.0 mol/L sucrose, for 1 min, followed by 3 min in diluent solution (DS; Vitrification kit, Kitazato, Japan), which contained 0.5 mol/L sucrose. They were then washed twice in washing solution 1 and 2 (WS1 and WS2; Vitrification kit, Kitazato, Japan) for 5 min each. The warmed embryos were then cultured for at least 2 h before post-warming evaluation. The temperature of the TS, WS2, and culture media were maintained at 37 °C. After warming, the embryos were checked for survival under an inverted microscope. They were immediately transferred after post-warming evaluation.

Blastocysts were transferred 5 days after transformation. Luteal phase support was provided from the day of transfer until the 10th week of gestation, with 90 mg/d vaginal P (8% Crinone, Merck, UK) and 20 mg/d oral dydrogesterone (Duphaston, Abbott, Netherlands).

### Outcome measures

The primary outcome measures were live birth rate (LBR) and clinical pregnancy rate (CPR). A clinical pregnancy was diagnosed when the serum hCG level reached > 20 IU/l at 2 weeks after transfer and the gestational sac was detected on ultrasound at 5–7 weeks after transfer. A live birth was defined as complete expulsion or extraction of a live baby after the 28th week of gestation. The secondary outcome measures included other pregnancy outcomes, maternal outcomes and neonatal outcomes. Maternal complications included gestational hypertension disorders (International Classification of Diseases (ICD) 10 codes O13-15), gestational diabetes mellitus (O24), premature abruption of membrane (O42), placenta previa (O44), placenta abruption (O45), and placenta accrete (O73). Neonatal outcomes included gestational week, preterm birth (PTB, < 37 weeks of gestation), very preterm birth (VPTB, < 32 weeks of gestation), birth weight (BW), low birth weight (LBW, < 2500 g), very low birthweight (VLBW, < 1500 g), small-for-gestational age (SGA, BW < 10th percentile of the average weight at the same gestational week), large-for-gestational age (LGA, BW > 90th percentile of the average weight at the same gestational week) [21], delivery mode, sex of newborn, congenital anomaly, pediatric intensive care unit (PICU) admission, and neonatal mortality.

### Statistical analysis

SAS 9.2 (SAS Inc., NC, USA) was used for statistical analysis. Continuous variables are presented as mean ± SD. Categorical variables are presented as number (percentage). Analysis of variance (ANOVA) and chi-square test were performed, as appropriate. Multiple comparisons between groups were corrected by the Bonferroni method. Multivariate logistic regression and multivariate general linear model were used to adjust for the potential confounders, with well-established variables as covariates, including age, type of infertility, IVF indications, antral follicle count (AFC), endometrial thickness, type of endometrial preparation, expansion stage, inner cell mass, and trophectoderm. Normal-weight women were used as the reference group for all the comparisons. Restricted cubic spline curves were used to evaluate the dose–response effect of BMI on the odds of live birth. Post hoc power analysis was used to evaluate the ability to find a significant difference. A power value ≥ 0.80 was considered as sufficient powered. A *P* value < 0.05 was considered statistically significant.

## Results

There were 10,252 frozen cycles with single blastocyst transfer, resulting in 5659 clinical pregnancies, 4426 live birth deliveries, and 4501 babies. The numbers of underweight, normal-BMI, overweight and obese women were 1127 (10.99%), 6925 (67.55%), 1810 (17.66%) and 390 (3.80%), respectively. The overall CPR was 55.20%, and the overall LBR was 43.14%.

Demographic and clinical features are presented in Table 1. Compared with the normal-weight women (31.93 ± 4.54 years old), the underweight women were younger (30.25 ± 3.94 years old, *P* < 0.0001), while the overweight women (32.80 ± 5.16 years old, *P* < 0.0001) were older. The proportion of primary infertility was higher in the underweight women (*P* < 0.0001). The duration of infertility (*P* < 0.0001) and baseline AFC (*P* < 0.0001) increased with increasing BMI. The AMH levels were comparable among groups. There was a significant difference in IVF indications among groups (*P* < 0.0001), with higher proportions of ovulation disorders and diminished ovarian reserve in the overweight and obese women. No difference was found among groups with respect to the number of oocytes retrieved and the distribution of IVF vs. ICSI. The proportion of good blastocysts (9.06 vs. 11.67%, *P* = 0.0286) was lower, and the proportions of fair (57.29 vs. 55.71%) and poor blastocysts (33.65 vs. 32.62%) were higher in the overweight women as compared to the normal controls. There was no difference in the distribution of endometrial preparation methods among groups. Compared with normal-BMI women (9.33 ± 1.54 mm), the underweight women (9.17 ± 1.42 mm, *P* = 0.0010) had thinner endometrium, while the obese women had thicker (9.41 ± 1.61 mm, *P* = 0.0009) endometrium.

**Table 1:** Demographic and clinical features

	BMI < 18.5	BMI 18.5–24	BMI 24–28	BMI ≥ 28	*P* value
No. of cycles	1127	6925	1810	390	
Age (years)	30.25 ± 3.94	31.93 ± 4.54	32.80 ± 5.16	31.74 ± 4.62	< 0.0001^ab^
BMI (kg/m^2^)	17.62 ± 0.74	21.11 ± 1.47	25.46 ± 1.08	29.85 ± 2.07	< 0.0001^abc^
Type of infertility, n (%)					< 0.0001^a^
Primary	823 (73.03)	4634 (66.92)	1177 (65.03)	258 (66.15)	
Secondary	304 (26.97)	2291 (33.08)	633 (34.97)	132 (33.85)	
Duration of infertility (years)	3.04 ± 1.89	3.27 ± 2.35	3.62 ± 2.60	3.79 ± 2.47	< 0.0001^bc^
AFC	11.60 ± 5.53	12.48 ± 5.80	13.26 ± 6.76	13.42 ± 6.52	< 0.0001^ab^
AMH (ng/ml)	5.07 ± 3.73	5.22 ± 3.78	5.44 ± 4.00	5.04 ± 3.89	0.1568
IVF indications, n (%)					< 0.0001^abc^
Tubal	406 (36.02)	2375 (34.30)	571 (31.55)	106 (27.18)	
Endometriosis	77 (6.83)	620 (8.95)	108 (5.97)	25 (6.41)	
Male	226 (20.05)	1242 (17.94)	256 (14.14)	57 (14.62)	
Ovulation disorders	203 (18.01)	1222 (17.65)	445 (24.59)	131 (33.59)	
Unknown factors	77 (6.83)	359 (5.18)	70 (3.87)	12 (3.08)	
DOR	102 (9.05)	901 (13.01)	304 (16.80)	49 (12.56)	
Others	36 (3.19)	206 (2.97)	56 (3.09)	10 (2.56)	
No. of oocytes retrieved	10.66 ± 4.60	11.14 ± 4.75	10.77 ± 4.80	10.61 ± 4.83	0.1070
Fertilization method, n (%)					0.7183
IVF	667 (59.18)	4216 (60.88)	1102 (60.88)	233 (59.74)	
ICSI	460 (40.82)	2709 (39.12)	708 (39.12)	157 (40.26)	
Embryo quality, n (%)					0.0286^b^
Good	128 (11.36)	808 (11.67)	164 (9.06)	32 (8.21)	
Fair	644 (57.14)	3858 (55.71)	1037 (57.29)	228 (58.46)	
Poor	355 (31.50)	2259 (32.62)	609 (33.65)	130 (33.33)	
Endometrial preparation, n (%)					0.8079
NC	67 (5.94)	383 (5.53)	103 (5.69)	15 (3.85)	
AC	946 (83.94)	5847 (84.43)	1527 (84.36)	339 (86.92)	
DR + AC	114 (10.12)	695 (10.04)	180 (9.94)	36 (9.23)	
Endometrial thickness (mm)	9.17 ± 1.42	9.33 ± 1.54	9.41 ± 1.61	9.60 ± 1.65	< 0.0001^ac^

^a^BMI < 18.5 vs. 18.5–24 kg/m^2^, ^b^BMI 24–28 vs. 18.5–24 kg/m^2^, ^c^BMI ≥ 28 vs. 18.5–24 kg/m^2^, *BMI* body mass index, *AFC* antral follicle count, *AMH* anti-Müllerian hormone, *NC* natural cycle, *AC* artificial cycle, *DR* down-regulation

Table 2 and Fig. 1 show pregnancy outcomes according to BMI categories. CPRs and LBRs were all comparable among groups. Miscarriage rate was higher in the obese group (27.51%, aOR = 1.453 (1.066–1.982)), compared with the normal-BMI group (20.91%). No difference was found in monozygotic twin-pregnancy or ectopic pregnancy rate among groups.

**Table 2:** Pregnancy outcomes according to BMI categories

	BMI < 18.5	BMI 18.5–24	BMI 24–28	BMI ≥ 28	*P* value
No. of cycles	1127	6925	1810	390	
CPR, n (%)	612 (54.30)	3841 (55.47)	977 (53.98)	229 (58.72)	0.3069
OR (95% CI)	0.954 (0.841–1.083)	REF	0.942 (0.849–1.045)	1.142 (0.928–1.405)	
aOR (95% CI)	0.880 (0.763–1.001)	REF	0.992 (0.891–1.104)	1.125 (0.908–1.393)	
LBR, n (%)	502 (44.54)	3011 (43.48)	747 (41.27)	166 (42.56)	0.2773
OR (95% CI)	1.044 (0.920–1.185)	REF	0.913 (0.822–1.015)	0.963 (0.784–1.184)	
aOR (95% CI)	0.935 (0.820–1.066)	REF	0.971 (0.871–1.083)	0.948 (0.765–1.174)	
Miscarriage rate per CP, n (%)	104 (16.99)	803 (20.91)	222 (22.72)	63 (27.51)	0.0037
OR (95% CI)	0.775 (0.619–0.970)	REF	1.112 (0.940–1.317)	1.436 (1.064–1.939)	
aOR (95% CI)	0.859 (0.682–1.081)	REF	1.066 (0.897–1.266)	1.453 (1.066–1.982)	
Ectopic rate per CP, n (%)	6 (0.98)	27 (0.70)	8 (0.82)	0 (0.00)	0.4985
OR (95% CI)	1.399 (0.575–3.402)	REF	1.166 (0.528–2.575)	NA	
aOR (95% CI)	1.523 (0.619–3.747)	REF	1.135 (0.511–2.520)	NA	
Twin pregnancy rate per CP, n (%)	16 (2.61)	110 (2.86)	21 (2.15)	7 (3.06)	0.6536
OR (95% CI)	0.911 (0.535–1.549)	REF	0.745 (0.465–1.195)	1.070 (0.492–2.324)	
aOR (95% CI)	0.879 (0.515–1.502)	REF	0.719 (0.444–1.166)	1.054 (0.484–2.296)	

*CPR* clinical pregnancy rate, *LBR* live birth rate, *CP* clinical pregnancy, *OR* odds ratio, *aOR* adjusted odds ratio, *REF* reference, *NA* not available

Maternal outcomes are presented in Table 3. Using the normal-BMI women as reference, the incidence of PTB was lower in the underweight group (6.97 vs. 11.19%, aOR = 0.611 (0.422–0.884)), while it was higher in the obese group (16.87 vs. 11.19%, aOR = 1.646 (1.068–2.536)). No difference was found in the incidence of VPTB among groups. The proportion of women needing a cesarean section was higher in the obese women (93.98% vs. 87.91%, aOR = 2.078 (1.083–3.987)). The overweight women had significantly higher incidences of gestational diabetes (6.56 vs. 3.82%, aOR = 1.744 (1.232–2.468)) and hypertension (4.42 vs. 2.32%, aOR = 1.822 (1.186–2.800)). The obese women had a higher incidence of gestational hypertension (4.82 vs. 2.32%, aOR = 2.138 (1.005–4.547)). No difference was found in the incidences of placenta accrete, placenta abruption, placenta previa, or premature rupture of membrane. Gestational age decreased with increasing BMI across groups (*P* < 0.0001).

**Table 3:** Maternal outcomes according to BMI categories

	BMI < 18.5	BMI 18.5–24	BMI 24–28	BMI ≥ 28	*P* value
No. of deliveries	502	3011	747	166	
Gestational age (week)	38.78 ± 1.60	38.52 ± 1.76	38.31 ± 1.87	38.25 ± 1.54	< 0.0001
SMD (95% CI)	0.257 (0.039 ~ 0.476)	REF	-0.198 (-0.383 ~ -0.012)	-0.273 (-0.636 ~ 0.090)	
PTB, n (%)	35 (6.97)	337 (11.19)	105 (14.06)	28 (16.87)	0.0002
aOR (95% CI)	0.611 (0.422–0.884)	REF	1.269 (0.997–1.615)	1.646 (1.068–2.536)	
VPTB, n (%)	4 (0.80)	41 (1.36)	12 (1.61)	1 (0.60)	0.5252
aOR (95% CI)	0.543 (0.192–1.531)	REF	1.253 (0.652–2.407)	0.434 (0.059–3.184)	
Cesarean section, n (%)	439 (87.45)	2647 (87.91)	673 (90.09)	156 (93.98)	0.0404
aOR (95% CI)	1.011 (0.755–1.353)	REF	1.233 (0.943–1.612)	2.078 (1.083–3.987)	
Gestational diabetes, n (%)	13 (2.59)	115 (3.82)	49 (6.56)	10 (6.02)	0.0012
aOR (95% CI)	0.689 (0.383–1.238)	REF	1.744 (1.232–2.468)	1.681 (0.859–3.288)	
Hypertension, n (%)	10 (1.99)	70 (2.32)	33 (4.42)	8 (4.82)	0.0035
aOR (95% CI)	0.854 (0.434–1.681)	REF	1.822 (1.186–2.800)	2.138 (1.005–4.547)	
PAI, n (%)	4 (0.80)	16 (0.53)	4 (0.54)	2 (1.20)	0.6466
aOR (95% CI)	1.362 (0.439–4.222)	REF	1.024 (0.337–3.111)	2.754 (0.618–12.277)	
Placenta abruption, n (%)	0 (0.00)	2 (0.07)	1 (0.13)	1 (0.60)	0.1315
aOR (95% CI)	NA	REF	NA	NA	
Placenta previa, n (%)	11 (2.19)	95 (3.16)	20 (2.68)	4 (2.41)	0.6105
aOR (95% CI)	0.727 (0.385–1.373)	REF	0.836 (0.511–1.367)	0.792 (0.287–2.189)	
PROM, n (%)	2 (0.40)	18 (0.60)	3 (0.40)	2 (1.20)	0.5997
aOR (95% CI)	0.677 (0.155–2.956)	REF	0.679 (0.199–2.318)	2.203 (0.502–9.659)	

*PTB* preterm birth, *VPTB* very preterm birth, *SMD* standardized mean difference, *aOR* adjusted odds ratio, *PAI* placenta accreta/increta, *PROM* premature rupture of membrane *REF* reference, *NA* not applicable

Table 4 shows the neonatal outcomes. The mean birthweight increased with increasing BMI (*P* = 0.0009). No difference was found in the incidence of LBW, VLBW or SGA among groups. Using normal-BMI group as reference, the incidences of macrosomia (4.90 vs. 8.65%, aOR = 0.544 (0.353–0.837)) and LGA (11.18 vs. 16.54%, aOR = 0.643 (0.477–0.866)) were lower in the underweight group. The incidences of macrosomia (12.93 vs. 8.65%, aOR = 1.596 (1.240–2.054)) and LGA (23.22 vs. 16.54%, aOR = 1.549 (1.270–1.890)) were higher in the overweight group. Higher incidences of macrosomia (14.88 vs. 8.65%, aOR = 1.880 (1.192–2.964)) and LGA (25.60 vs. 16.54%, aOR = 1.764 (1.218–2.555)) were also found in the obese group. There was no difference in the incidences of congenital anomaly, PICU admission or neonatal death.

**Table 4:** Neonatal outcomes according to BMI categories

	BMI < 18.5	BMI 18.5–24	BMI 24–28	BMI ≥ 28	*P* value
No. of neonates	510	3065	758	168	
Birth weight (kg)	3286.46 ± 449.02	3333.16 ± 524.42	3395.18 ± 564.44	3408.11 ± 533.42	0.0009
SMD (95% CI)	-45.69 (-111.17 ~ 19.79)	REF	66.24 (10.70 ~ 121.78)	75.58 (-33.78 ~ 184.93)	
LBW, n (%)	17 (3.33)	163 (5.32)	46 (6.07)	7 (4.17)	0.1555
aOR (95% CI)	0.612 (0.361–1.037)	REF	1.064 (0.748–1.514)	0.823 (0.378–1.791)	
VLBW, n (%)	3 (0.59)	25 (0.82)	4 (0.53)	0 (0.00)	0.5422
aOR (95% CI)	0.613 (0.182–2.064)	REF	0.677 (0.233–1.964)	NA	
Macrosomia, n (%)	25 (4.90)	265 (8.65)	98 (12.93)	25 (14.88)	< 0.0001
aOR (95% CI)	0.544 (0.353–0.837)	REF	1.596 (1.240–2.054)	1.880 (1.192–2.964)	
SGA, n (%)	28 (5.49)	171 (5.58)	40 (5.28)	10 (5.95)	0.9829
aOR (95% CI)	0.940 (0.616–1.434)	REF	0.931 (0.647–1.339)	1.123 (0.580–2.176)	
LGA, n (%)	57 (11.18)	507 (16.54)	176 (23.22)	43 (25.60)	< 0.0001
aOR (95% CI)	0.643 (0.477–0.866)	REF	1.549 (1.270–1.890)	1.764 (1.218–2.555)	
Male neonate, n (%)	287 (56.27)	1742 (56.84)	431 (56.86)	89 (52.98)	0.7992
aOR (95% CI)	0.983 (0.808–1.195)	REF	1.005 (0.851–1.186)	0.874 (0.633–1.207)	
CA, n (%)	1 (0.20)	24 (0.78)	3 (0.40)	2 (1.19)	0.2760
aOR (95% CI)	0.250 (0.033–1.878)	REF	0.541 (0.160–1.825)	1.574 (0.361–6.856)	
PICU admission, n (%)	0 (0.00)	4 (0.13)	4 (0.53)	1 (0.60)	0.0670
aOR (95% CI)	NA	REF	4.085 (0.997–16.571)	4.456 (0.490–40.495)	
Neonatal death, n (%)	0 (0.00)	7 (0.23)	2 (0.26)	1 (0.60)	0.5212
aOR (95% CI)	NA	REF	1.069 (0.218–5.254)	2.563 (0.309–21.238)	

There were 8, 54, 11, and 2 monozygotic twin-live birth in the groups of BMI < 18.5, 18.5–24, 24–28, ≥ 28 kg/m^2^, respectively. *SMD* standardized mean difference, *aOR* adjusted odds ratio, *LBW* low birth weight, *VLBW* very low birth weight, *SGA* small-for-gestational age, *LGA* large-for-gestational age, *CA* congenital anomaly, *PICU* pediatric intensive care unit, *REF* reference, *NA* not applicable

The pregnancy outcomes of an additional cohort with day 3 embryo transfer are shown in Additional Table 1. The LBR was higher in the underweight group compared with that in the normal-BMI group (23.00 vs. 14.21%, aOR = 1.498 (1.025–2.189)). The LBRs in the groups of overweight and obesity were both similar with that in the normal-BMI group.

## Discussion

This study included 10,252 cycles with single blastocyst transfer. No significant effect of BMI on CPR or LBR was observed among the women with different BMI categories. However, there was a higher miscarriage rate in the obese women compared with the normal-weight women. BMI had effects on maternal and neonatal outcomes, in terms of gestational age, obstetric complication, birth weight, and the type of delivery. Compared with the normal controls, the underweight women had lower incidences of PTB, macrosomia and LGA. The overweight women had higher incidences of gestational diabetes, hypertension, macrosomia and LGA. The obese women had higher incidences of PTB, gestational hypertension, cesarean delivery, macrosomia and LGA.

### Pregnancy outcomes

When BMI was evaluated as a continuous variable, the multivariate logistic model showed no significant effect of BMI on the chance of live birth. The OR increased 0.01 with increasing a unit of BMI (aOR = 1.01 (0.99–1.02)). Moreover, there was no inflection point in the restricted cubic spline curve. Therefore, we found no BMI cutoff, which is associated with a significant increase or decrease of the chance of live birth (Additional Fig. 2).

#### Underweight

Literatures reporting IVF outcomes of underweight women are relatively scarce. A retrospective study reported that low BMI was associated with a reduced LBR and an increased miscarriage rate compared with normal-BMI [22]. Similarly, a national study found slight but statistically significant lower chances of both clinical pregnancy and live birth in underweight women, compared with the normal controls [12]. In contrast, a large study by Zhang et al. found limited impact of low BMI on CPR or LBR [23]. Our study also found no significant difference in CPR or LBR between the underweight and normal-weight groups. This discrepancy is possibly attributed to the variations in methodology. The two studies reporting worse CPR and/or LBR in underweight women were both on fresh cycles, while the study by Zhang et al. and our study were on frozen cycles. In addition, previous studies included both cycles with cleavage and blastocyst-stage embryo transfer, and the number of embryos transferred ranged from 1 to > 3. We included a uniform cohort, and all cycles in our study were transferred with a single blastocyst.

To further elucidate the effect of embryo stage on pregnancy outcomes of underweight women, we included another cohort of women with a single cleavage-stage embryo transfer (Additional Table 1). The LBR in the underweight group (23.00%) was higher than those in the normal-BMI (14.21%), overweight (13.37%) and obese (12.50%) groups. Such advantage of underweight women over women with other BMI categories disappeared, when they were all transferred with blastocysts. These results suggest that underweight women may have better cleavage-stage embryos with higher developing potential. Their embryos are more likely to reach blastocyst-stage, and subsequently to result in a live birth. Indeed, the relationship between BMI and blastocyst formation rate has been reported [24]. Blastocyst culture serves as an in vitro screening tool for embryos with better competence, and thus equalizes pregnancy outcomes among women with different BMI categories. This is partially in agreement with our preliminary hypothesis.

#### Overweight and obesity

Prior studies assessing the association of excess weight and IVF outcomes showed inconsistent results, possibly due to the great variations across studies, in terms of population, definition, sample size and methodology. Some studies showed a decline in the chance of pregnancy with increased BMI [5, 12]; however, other studies did not find an association between BMI and IVF outcomes. To further complicate this issue, randomized studies on preconception weight loss also showed conflicting results [25–27]. Our study showed that the overweight and obese women had similar CPRs and LBRs to the normal controls. One reason for this discrepancy is that the definitions of overweight and obesity were different across studies. We used the BMI of 24 and 28 kg/m^2^ according to the Chinese criteria, which has been demonstrated to be more suitable for Chinese population. Intriguingly, we also classified the cohort with the WHO classification, and the results were similar (Additional Table 2). Another reason is the heterogeneity of ethnicity. Chinese women are relatively short and lean than western women. We further performed a subgroup analysis in the obese women (Additional Table 3). The mean and median of BMI in the obese group were 29.85 ± 2.07 kg/m^2^ and 29.12 (28.00–39.04) kg/m^2^. Patients were divided into 3 subgroups: 28–30, 30–35, and ≥ 35 kg/m^2^. The proportions of women with a BMI of 28–30, 30–35, ≥ 35 kg/m^2^ were 64.87, 31.03 and 4.10%, respectively. Only 16 out of 390 women were severely obese (35–40 kg/m^2^). No woman was extremely obese (≥ 40 kg/m^2^). These data indicate that even in the Chinese women diagnosed as obesity, most of them had a BMI just over 28 kg/m^2^, and their disorders of glucose/lipid metabolism might be relatively mild. Indeed, a BMI of ≥ 35 kg/m^2^ has been proposed as an extremity of clinical value to decrease pregnancy and live birth rates [28].

Another difference across studies is the type of cycle. Many studies showing negative effects of BMI were based on fresh cycles [6, 12, 29]. In contrast, studies on frozen cycles showed neutral effect of BMI on pregnancy outcomes [13, 14, 30]. Our study was on frozen cycles, and the result was in consistent with the latter ones. It is well established that in a fresh cycle, gonadotropins therapy results in supraphysiologic hormone level, such as estradiol, and thereby alter the implantation window. A frozen transfer protocol does not use gonadotropins, and thus improves IVF success. Women with increased BMI require higher doses and longer duration of stimulation. This might explain why worse pregnancy outcomes was observed by studies on fresh cycles, but not in studies with frozen cycles.

The stage from cleavage-stage embryo to blastocyst is critical for embryonic development. This stage is also developmental susceptibility, and thus excess weight may have significantly harmful effects on embryonic health during this period. Therefore, we hypothesized that blastocyst- vs. cleavage-stage embryo transfer would improve the IVF outcomes in overweight/obese women. However, our results do not support this hypothesis. In the cohort with blastocyst transfer, there was no difference in the LBR among the groups of overweight, obesity and normal-BMI. In the additional cohort with day 3 embryo transfer, the LBRs in the groups of overweight and obesity were also not significantly lower than that in the normal-BMI group. The failed demonstration of our hypothesis indicates that the nature of the impact of BMI on embryo development and pregnancy is more complicated than we preliminarily thought, which warrants further elucidations.

In this study, the obese women had a higher risk of miscarriage (27.51 vs. 20.91%, aOR = 1.453 (1.066–1.982)) relative to the normal-BMI women. This result is in line with prior studies reporting higher miscarriage rates in women with excess weight. The adverse effects of obesity on pregnancy outcomes are multifaceted. A possible mechanism is that obesity may affect oocyte quality. This effect may persist during early embryo development, and result in aneuploidy/mosaicism or disorders in developmental dynamics. Indeed, animal studies have reported that obesity alters maternal metabolic environment, and exerts a long-term effect on oocytes and subsequent embryos [17]. However, studies on donor/recipient cycles found that recipient LBRs decreased, and miscarriage rates increased with increasing BMIs of donors [31, 32]. This result suggests that an endometrial/placental factor may also affect pregnancy in women with excess weight. In addition, endocrine abnormality is also an important factor. Obesity is related to insulin resistance, metabolic disturbance and chronic inflammation [33]. This may further affect the maintenance of pregnancy. The mechanism underlying the relationship of BMI and miscarriage needs further evaluation.

### Maternal and neonatal outcomes

Prior studies on general population (including both natural and IVF conception) have provided evidence of deleterious effects of increased BMI on both maternal and neonatal outcomes. A large population-based study observed worse maternal and perinatal outcomes, in terms of gestational diabetes, preeclampsia, cesarean delivery, macrosomia, low Apgar score and stillbirth [1]. In addition, obesity has been linked with increased risks of congenital anomalies, such as neural tube defects, spina bifida, and cardiovascular anomalies [9]. Mothers with a higher BMI are also at increased risk of deliveries affected by fetal, perinatal and neonatal deaths [10].

Clinical data on the association between BMI and maternal/neonatal outcomes in the setting of IVF are lacking. A large national study by Kawwass et al. reported only two delivery outcomes, PTB and LBW [12]. Intriguingly, they found increased incidences of PTB and LBW in the underweight mothers. On the contrary, we found that the incidence of PTB was lower in the underweight mother, compared with the normal controls (6.97 vs. 11.19%), and the incidence of LBW in the underweight mothers was similar to that in the normal controls, but the incidences of macrosomia (4.90 vs. 8.65%) and LGA (11.18 vs. 16.54%) were both lower in the underweight mothers. As for the mothers with excess weight, Kawwass et al. reported higher incidences of both PTB and LBW. We did not observe such difference in LBW. However, we did find a higher incidence of PTB in the obese women. Additionally, we found higher incidences of macrosomia and LGA in overweight and obese mothers.

In this study, the obese mothers were more likely to need a cesarean delivery. This result is consistent with prior studies on general population [1]. In obstetric scenarios, a labor is often complicated by the obesity. The duration of the first stage of labor is longer, and the progression of the latent phase is slower in obese women [34]. Moreover, obese mothers are more likely to have comorbidities, and to deliver a greater baby. In addition, many pregnant women in China, who have achieved a pregnancy via IVF, prefer cesarean delivery, because they believe their fetuses are very precious. These conditions result in a higher cesarean delivery rate in obese women.

Regarding obstetric complications, the overweight women had significantly higher risks of gestational diabetes and hypertension. The obese women had a higher risk of gestational hypertension. No statistically significant difference was found in placental disorders, including placenta accrete, placenta abruption, placenta previa and membrane rupture. This result suggests that excess weight may exert adverse effects mainly via systemic metabolic pathways, but not via placenta.

### Clinical implications

Many underweight women are recommended to gain weight before initiation of IVF. Our results do not support this strategy. According to our results, underweight women have a similar or even better outcomes. This result indicates that preconception undernutrition status does not affect pregnancy, maternal or neonatal outcomes.

Some fertility centers recommend prohibiting IVF in women with a BMI of 25–40 kg/m^2^. However, some literatures argued that it is unreasonable and unethical not to offer IVF to obese women [35, 36]. In our study, although women with excess weight performed well with respect to CPR and LBR, they had significant higher risks of miscarriage, PTB, macrosomia, LGA, cesarean delivery, gestational diabetes, and gestational hypertension. Therefore, well informed consent is needed before IVF in overweight and obese women. Weight reduction therapy is recommended when it is necessary.

This study also demonstrated the efficacy of protocols of FET and blastocyst culture. Compared with prior studies reporting lower LBRs in fresh cycles, our study suggests that FET may reduce the adverse effect of excess weight on pregnancy outcomes. In this study, the overall LBR after blastocyst transfer was 43.14%, but the overall LBR after day 3 embryo transfer was only 14.68%. Blastocyst culture can help to screen embryos with better competence, and this may alleviate negative effects of increased BMI.

### Strengths and limitations

A strength of this study is its large sample size, a total of 10,252 cycles were analyzed. Another strength is that it provides clinical evidence of pregnancy outcomes across BMI categories in Chinese population. In addition, this was a mono-center study, therefore consistent and stable practices (guided by the Standard Operation Protocols (SOP) of ISO9001) were performed during the study period. We only included FET cycles with single blastocyst transfer. This uniform cohort is good for the analysis.

Admittedly, there are some limitations in our study. First, there might be selection bias and confounders due to the retrospective nature. Nevertheless, multivariate models were used to adjust for well-established covariates. Second, the measuring of BMI may be another potential confounder. As discussed by previous studies, patients’ BMI values might be underestimated based on their self-reported data. In this study, we measured patients’ weight and height, and calculated their BMIs. The value of BMI at transfer may vary from that measured before oocytes retrieval. At our center, we measure patients’ BMIs multiple times during their fresh cycles, and re-measure them at the beginning of frozen cycles. If a patient has an obvious varied BMI, which results in a change of diagnosis (for example, from normal-weight to overweight), she will be counselled to control her diet and weight. Such patients were excluded from this study. Third, this study was underpowered to detect differences in several obstetric and newborn outcomes. A further prospective study with sufficient power can confirm our findings. Fourth, BMI is a marker of general obesity. Waist circumference and waist to hip ratio are anthropometric measures of central obesity. It has been reported that they are both associated with the chance of live birth, and their predicting value might be above BMI [4, 33]. A combination use of BMI, waistline and waist to hip ratio may better elucidate the association of obesity and pregnancy outcomes. Finally, this study is on Chinese women. Therefore, the results should be interpreted with caution when generalized to other ethnicities.

## Conclusions

BMI has no significant effect on the chance of pregnancy or live birth, but obesity increases the risk of miscarriage. Underweight is associated with lower risks of PTB, macrosomia and LGA. Overweight impairs both maternal and neonatal outcomes, in terms of gestational diabetes, hypertension, macrosomia, and LGA. Obese is associated with higher risks of PTB, gestational hypertension, macrosomia and LGA. A further study with greater sample size of obese women is needed.

## Supplementary Information

**Additional file 1.** Flow chart of patient selection

**Additional file 2.** Restricted cubic spline curves of the association between BMI and live birth

**Additional file 3.** Pregnancy outcomes after transfer with a single day 3 embryo

**Additional file 4.** Pregnancy outcomes according to the WHO classification

**Additional file 5.** Pregnancy outcomes in the BMI category of ≥28 kg/m^2^

## Data Availability

All data generated or analyzed during this study are included in this published article. Further inquiries can be directed to the corresponding author.

## Abbreviations

BMI: Body mass index
IVF: In vitro fertilization
FET: Frozen-thawed embryo transfer
PGT: Preimplantation genetic testing
NC: Natural cycle
AC: Artificial cycle
GnRH-a: Gonadotropin releasing hormone-agonist
FSH: Follicle stimulating hormone
SC: Subcutaneous
AFC: Antral follicle count
AMH: Anti-Müllerian hormone
E2: Estradiol
P: Progesterone
LH: Luteinizing hormone
hCG: Human chorionic gonadotropin
ICSI: Intracytoplasmic sperm injection
CPR: Clinical pregnancy rate
LBR: Live birth rate
PTB: Preterm birth
VPTB: Very preterm birth
LBW: Low birth weight
VLBW: Very low birthweight
SGA: Small-for-gestational age
LGA: Large-for-gestational age
PICU: Pediatric intensive care unit
