# Supplementary figures and images for: Body mass index is associated with miscarriage rate and perinatal outcomes in cycles with frozen-thawed single blastocyst transfer: a retrospective cohort study
Source: BMC Pregnancy Childbirth. 2022 Feb 11;22:118. doi: 10.1186/s12884-022-04443-2 (PMC8840631; doi:10.1186/s12884-022-04443-2)

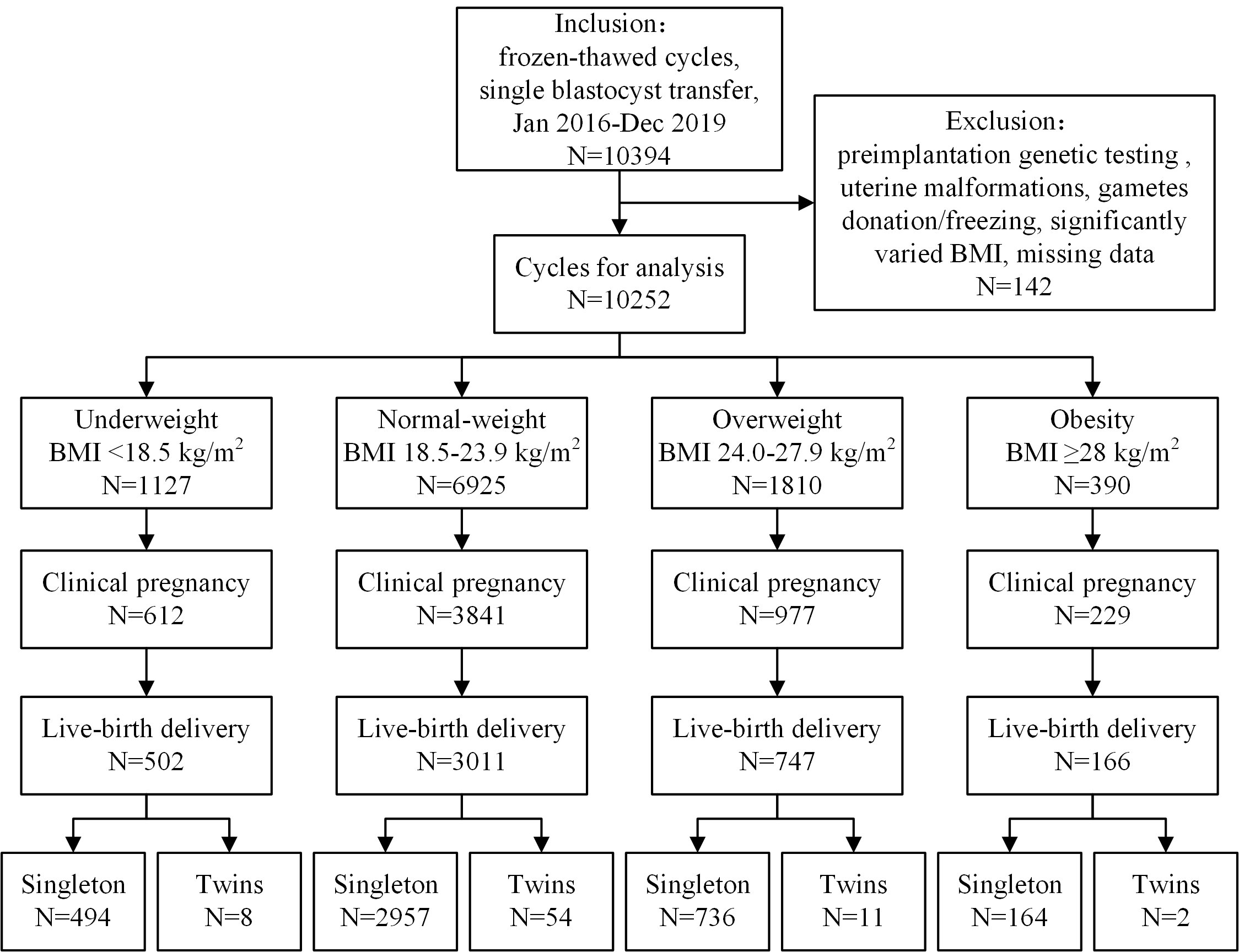

Supplement: Supplementary file 1 — Additional file 1. Flow chart of patient selection [file 12884_2022_4443_MOESM1_ESM.tif]

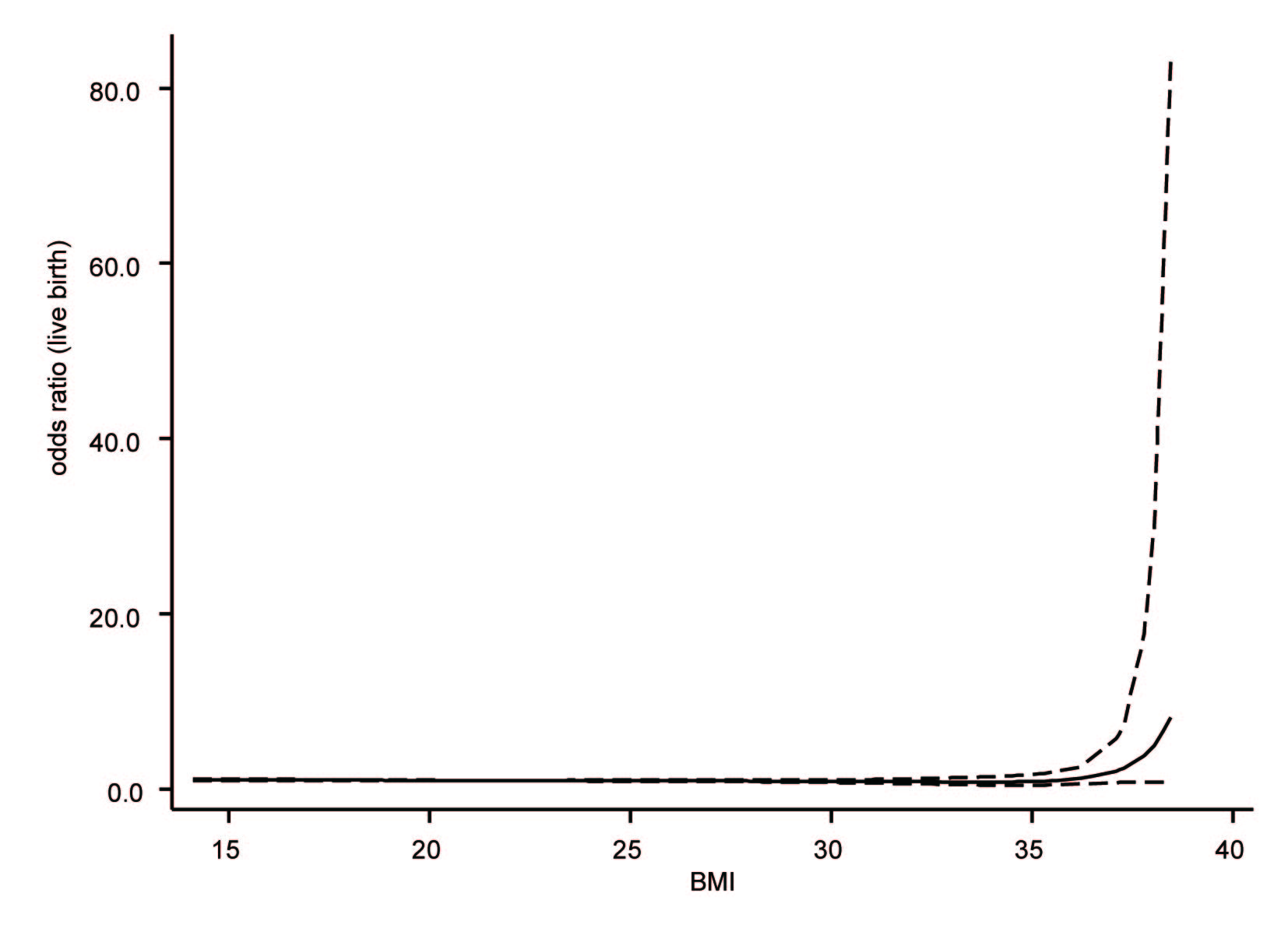

Supplement: Supplementary file 3 — Additional file 3. Pregnancy outcomes after transfer with a single day 3 embryo [file 12884_2022_4443_MOESM3_ESM.jpg]
