# Supplementary material for: Body mass index is associated with miscarriage rate and perinatal outcomes in cycles with frozen-thawed single blastocyst transfer: a retrospective cohort study
Source: BMC Pregnancy Childbirth. 2022 Feb 11;22:118. doi: 10.1186/s12884-022-04443-2 (PMC8840631; doi:10.1186/s12884-022-04443-2)
Supplement: Supplementary file 2 — Additional file 2. Restricted cubic spline curves of the association between BMI and live birth [file 12884_2022_4443_MOESM2_ESM.docx]

Additional Table 1 Pregnancy outcomes after transfer with a single day 3 embryo

|  | BMI <18.5 | BMI 18.5-24 | BMI 24-28 | BMI ≥28 | P value |
| --- | --- | --- | --- | --- | --- |
| N | 200 | 1611 | 546 | 96 |  |
| CPR, n (%)  OR (95% CI)  aOR (95% CI) | 58 (29.00)  1.488 (1.072-2.065)  1.241 (0.878-1.754) | 347 (21.54)  REF  REF | 107 (19.60)  0.888 (0.697-1.131)  1.036 (0.802-1.339) | 21 (21.88)  1.020 (0.620-1.679)  0.997 (0.585-1.699) | 0.0524  0.0169 |
| LBR, n (%)  OR (95% CI)  aOR (95% CI) | 46 (23.00)  1.803 (1.261-2.578)  1.498 (1.025-2.189) | 229 (14.21)  REF  REF | 73 (13.37)  0.931 (0.702-1.237)  1.099 (0.814-1.483) | 12 (12.50)  0.862 (0.463-1.6040)  0.774 (0.396-1.514) | 0.0060  0.0011  0.0343 |
| MR per CP, n (%)  OR (95% CI)  aOR (95% CI) | 11 (18.97)  0.518 (0.259-1.038)  0.585 (0.280-1.219) | 108 (31.12)  REF  REF | 31 (28.97)  0.903 (0.561-1.452)  0.883 (0.537-1.450) | 9 (42.86)  1.660 (0.679-4.056)  2.140 (0.812-5.643) | 0.1519 |
| ER per CP, n (%)  OR (95% CI)  aOR (95% CI) | 1 (1.72)  0.591 (0.074-4.708)  NA | 10 (2.88)  REF  NA | 3 (2.80)  0.972 (0.263-3.599)  NA | 0 (0.00)  NA  NA | 0.8368 |

Note:

CPR: clinical pregnancy rate; LBR: live birth rate; MR per CP: miscarriage rate per clinical pregnancy; ER per CP: ectopic pregnancy per CP; OR: odds ratio; aOR: adjusted odds ratio; REF: reference; NA: not available
