# Supplementary material for: Body mass index is associated with miscarriage rate and perinatal outcomes in cycles with frozen-thawed single blastocyst transfer: a retrospective cohort study
Source: BMC Pregnancy Childbirth. 2022 Feb 11;22:118. doi: 10.1186/s12884-022-04443-2 (PMC8840631; doi:10.1186/s12884-022-04443-2)
Supplement: Supplementary file 4 — Additional file 4. Pregnancy outcomes according to the WHO classification [file 12884_2022_4443_MOESM4_ESM.docx]

Additional Table 2 Pregnancy outcomes according to the WHO classification

|  | BMI <18.5 | BMI 18.5-25 | BMI 25-30 | BMI ≥30 | P value |
| --- | --- | --- | --- | --- | --- |
| N | 1127 | 7632 | 1356 | 137 |  |
| CPR, n (%) | 612 (54.30) | 4194 (54.95) | 776 (57.23) | 77 (56.20) | 0.4131 |
| LBR, n (%) | 502 (44.54) | 3284 (43.03) | 582 (42.92) | 58 (42.34) | 0.8010 |
| MR per CP, n (%) | 104 (16.99) | 882 (21.03) | 187 (24.10) | 19 (24.68) | 0.0117 |
| ER per CP, n (%) | 6 (0.98) | 28 (0.67) | 7 (0.90) | 0 (0.00) | 0.6486 |
