# Supplementary material for: Body mass index is associated with miscarriage rate and perinatal outcomes in cycles with frozen-thawed single blastocyst transfer: a retrospective cohort study
Source: BMC Pregnancy Childbirth. 2022 Feb 11;22:118. doi: 10.1186/s12884-022-04443-2 (PMC8840631; doi:10.1186/s12884-022-04443-2)
Supplement: Supplementary file 5 — Additional file 5. Pregnancy outcomes in the BMI category of ≥28 kg/m2 [file 12884_2022_4443_MOESM5_ESM.docx]

Additional Table 3 Pregnancy outcomes in the BMI category of ≥28 kg/m^2^

|  | BMI 28-30 | BMI 30-35 | BMI ≥35 | P value |
| --- | --- | --- | --- | --- |
| N | 253 (64.87) | 121 (31.03) | 16 (4.10) |  |
| CPR, n (%) | 152 (60.08) | 64 (52.89) | 13 (81.25) | 0.0729 |
| LBR, n (%) | 108 (42.69) | 48 (39.67) | 10 (62.50) | 0.2212 |
| MR per CP, n (%) | 45 (29.61) | 17 (26.56) | 3 (23.08) | 0.8203 |
| ER per CP, n (%) | 0 (0.00) | 0 (0.00) | 0 (0.00) | NA |
